# *Nfkb1*/p50 and mammalian aging

**DOI:** 10.18632/oncotarget.3405

**Published:** 2015-01-30

**Authors:** Bakhtiar Yamini

**Affiliations:** Department of Surgery, Section of Neurosurgery, The University of Chicago, Chicago, IL, USA

Aging is a progressive process that involves a combination of genetic and acquired factors that ultimately cause loss of tissue homeostasis and death. Among the pathways that modulate aging, NF-κ B has been shown to play a central role [1]. Interestingly, the majority of studies examining NF-κB and aging suggest that this transcription factor promotes aging. However, as was shown with the role of NF-κB in carcinogenesis and the response to DNA damage, NF-κB proteins often have antagonistic effects in the regulation of cellular processes. In this regard, we recently demonstrated that the p50 (NF-κB1) subunit actually attenuates mammalian aging [2]. These opposing findings regarding NF-κB and aging are likely in part explained by the subunit specific nature of the NF-κB response.

The five NF-κB proteins, p50 (NF-κB1, p105), p52 (NF-κB2, p100), p65 (relA), c-rel, and relB modulate gene expression as dimers. In examining the role of p50 in the response to DNA damage, we reported that this subunit is required for genome maintenance in the setting of replication stress (RS) induced during the cell cycle [3]. This observation raised the question of whether loss of p50/NF-κB1 in animals results in the development of chronic disease. Although mice deleted of *Nfkb1*, the parental protein of p50, were described almost 2 decades ago, they were reported to have a normal lifespan up to 1 year with the only defects being in innate and adaptive immunity [4]. Nevertheless, recent studies have found that these mice prematurely develop age-related findings that involve the central nervous system (CNS) [5].

To better examine the phenotypic consequence of loss of this subunit, we obtained *Nfkb1*^−/−^ animals that had been backcrossed to C57BL6 mice for 12 generations and interbred them with wildtype animals to obtain single strain littermates [2]. Animals were then followed in a pathogen-free environment over an extended period of time. Although *Nfkb1*^−/−^ mice are slightly small, they are otherwise identical to their littermates for the first six months of age. However, by 12 months, *Nfkb1*^−/−^ animals have a higher incidence of multiple observable age-related characteristics and also develop kyphosis and osteoporotic changes at a significantly higher rate than age-matched wildtype animals. These premature age-related changes are underlined by a decrease in the lifespan of *Nfkb1*^−/−^ mice compared to wildtype and heterozygous littermates. To examine the histological changes associated with loss of *Nfkb1*, tissue samples were isolated from age-matched animals. Consistent with our prior observation that loss, or site-specific mutation, of p50 in cells results in an increase in histone H2AX phosphorylation (γH2AX) and senescence [3], *Nfkb1*^−/−^ mice have reduced apoptotic cell number and increased γH2AX staining in their brain tissue compared to control.

The premature aging of *Nfkb1*^−/−^ mice raised the question of whether loss of this subunit is associated with physiological aging. We therefore harvested tissue from a series of young and old wildtype mice. While we corroborated the well known finding that aging leads to an increase in NF-κB DNA binding [1], we also noted that in both aged tissue and serially passaged primary mouse embryonic fibroblasts (MEFs), there is an increase in the expression of p52 protein. Most remarkably, however, we found that the DNA-bound NF-κB dimer composition changes with age such that while p50/p65 makes up the DNA-bound dimer in young tissue, in old tissue p52 replaces p50. This latter observation suggests that the NF-κB dimer in physiologically aged tissue is similar to that in *Nfkb1*^−/−^ mice in that p50 is lost and replaced by p52 [6]. Despite the functional redundancy of NF-κB subunits, p52 does not mediate the response to RS in the same manner as p50 resulting in an increase in cellular senescence [3, 7].

In sum, we find that loss of p50/*Nfkb1* leads to a decrease in cellular apoptosis and an increase in senescence that is associated with premature animal aging. Interestingly, the increase in aging is not associated with an increase in tumor formation possibly because the increased cellular senescence acts to suppress tumor formation. p50 is ideally situated to modulate the response to a universal process such as RS because it is not only constitutively produced and found in virtually all tissues, but because it is also the primary DNA-bound NF-κB subunit present at baseline. While it is difficult to definitively say whether the loss of p50 DNA binding with age is a cause, or consequence, of aging, our data nevertheless emphasize the importance of p50 to aging and indicate that further examination of this subunit is warranted.
